# Differentially expressed microRNAs in lung adenocarcinoma invert effects of copy number aberrations of prognostic genes

**DOI:** 10.18632/oncotarget.24070

**Published:** 2018-01-08

**Authors:** Tomas Tokar, Chiara Pastrello, Varune R. Ramnarine, Chang-Qi Zhu, Kenneth J. Craddock, Larrisa A. Pikor, Emily A. Vucic, Simon Vary, Frances A. Shepherd, Ming-Sound Tsao, Wan L. Lam, Igor Jurisica

**Affiliations:** ^1^ Princess Margaret Cancer Centre, University Health Network, Toronto, Canada; ^2^ The Vancouver Prostate Centre, Vancouver General Hospital, Vancouver, Canada; ^3^ Department of Integrative Oncology, British Columbia Cancer Research Centre, Vancouver, Canada; ^4^ Mathematical Institute, University of Oxford, Oxford, United Kingdom; ^5^ Faculty of Mathematics, Physics and Informatics, Comenius University, Bratislava, Slovakia; ^6^ Department of Medical Biophysics, University of Toronto, Toronto, Canada; ^7^ Department of Laboratory Medicine and Pathobiology, University of Toronto, Toronto, Canada; ^8^ Department of Computer Science, University of Toronto, Toronto, Canada; ^9^ Institute of Neuroimmunology, Slovak Academy of Sciences, Bratislava, Slovakia

**Keywords:** lung adenocarcinoma, copy number aberrations, microRNA, gene regulatory network, prognostic signature

## Abstract

In many cancers, significantly down- or upregulated genes are found within chromosomal regions with DNA copy number alteration opposite to the expression changes. Generally, this paradox has been overlooked as noise, but can potentially be a consequence of interference of epigenetic regulatory mechanisms, including microRNA-mediated control of mRNA levels.

To explore potential associations between microRNAs and paradoxes in non-small-cell lung cancer (NSCLC) we curated and analyzed lung adenocarcinoma (LUAD) data, comprising gene expressions, copy number aberrations (CNAs) and microRNA expressions. We integrated data from 1,062 tumor samples and 241 normal lung samples, including newly-generated array comparative genomic hybridization (aCGH) data from 63 LUAD samples.

We identified 85 “paradoxical” genes whose differential expression consistently contrasted with aberrations of their copy numbers. Paradoxical status of 70 out of 85 genes was validated on sample-wise basis using The Cancer Genome Atlas (TCGA) LUAD data. Of these, 41 genes are prognostic and form a clinically relevant signature, which we validated on three independent datasets. By meta-analysis of results from 9 LUAD microRNA expression studies we identified 24 consistently-deregulated microRNAs. Using TCGA-LUAD data we showed that deregulation of 19 of these microRNAs explains differential expression of the paradoxical genes.

Our results show that deregulation of paradoxical genes is crucial in LUAD and their expression pattern is maintained epigenetically, defying gene copy number status.

## INTRODUCTION

Integration of array comparative genomic hybridization (aCGH) with mRNA microarray data has revealed significant associations between occurrence of copy number aberrations (CNAs) and differential gene expression in diverse cancers [1–7]. However, significantly downregulated genes have been often found to reside within chromosomal regions with increased number of copies (gains) and *vice versa*, creating a paradoxical signal. For example, Phillips *et al.* reported that 14% of the genes downregulated in prostate cancer reside within regions of DNA copy number gains, and approximately 9% of upregulated ones reside in regions of DNA copy number loss [1]. Usually, this paradox is ignored as a noise, but can potentially be a consequence of interference of other regulatory mechanisms controlling mRNA transcription [8].

In recent years, the cancer research community has investigated how epigenetic regulators, known as microRNAs (miRNAs), form prognostic signatures and affect regulatory pathways that can lead to tumorigenesis. miRNAs are short non-coding RNAs that regulate the translation of mRNA by serving as guide molecules in mRNA silencing, mediated by various associated proteins [9]. Targeting most protein-coding transcripts [10], miRNAs are involved in diverse biological processes, including development and homeostasis [11, 12]. Moreover, growing evidence implicates miRNAs as factors associated with major human pathologies, including cancer [13–16].

In 2012 approximately 13% of all new cancer cases worldwide were cancers of lung (and bronchus), making lung cancer one of the most frequent cancer type (surpassed only by breast cancer in women) [17]. Despite smoking cessation, and advances in detection and treatment, lung cancer remains the main cause of cancer-related death worldwide for both men and women [18]. With nearly 160,000 deaths annually it kills more people than other common cancers combined, including colon, breast and prostate [18]. The most common type of lung cancer is lung adenocarcinoma (LUAD), comprising approximately 45% of all lung cancer cases [19, 20].

**Figure 1:**
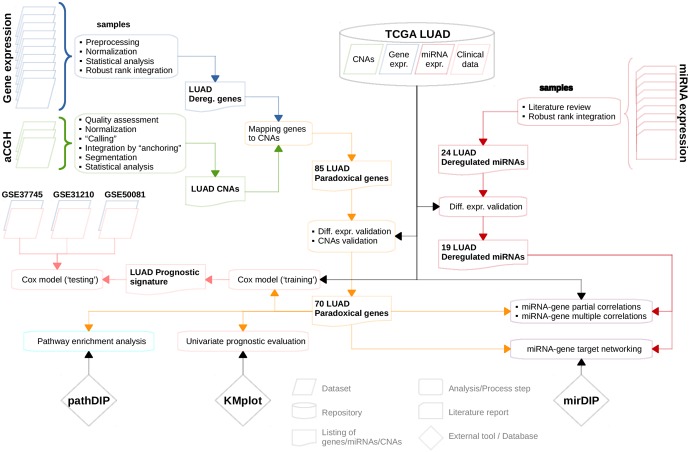
Flowchart depicting sequence of analyses/computational steps as performed and datasets as used For more details see Materials and Methods section.

In this paper, we integratively analyzed gene expression and CNA data from 12 publicly available LUAD datasets, and new CNA data obtained from 63 LUAD samples profiled at our institution (Figure 1). By combining and analyzing data from 1,062 tumor tissue samples and 241 normal samples we identified genes whose differential expression consistently was in contrast to aberrations of their copy numbers. Paradoxical status of these genes was then validated on the sample level, using TCGA LUAD gene expression and CNA data. Furthermore, to assess whether the paradoxical expression patterns were caused by epigenetic disruptions in lung tumors, we compiled miRNA expression data from 406 LUAD samples and 321 normal lung samples. Using miRNA:gene associations from mirDIP [21] and the measure of co-expression, we showed that this paradox can be explained by 19 miRNAs consistently deregulated in LUAD.

## RESULTS

### Identification of the paradoxical genes

**Figure 2:**
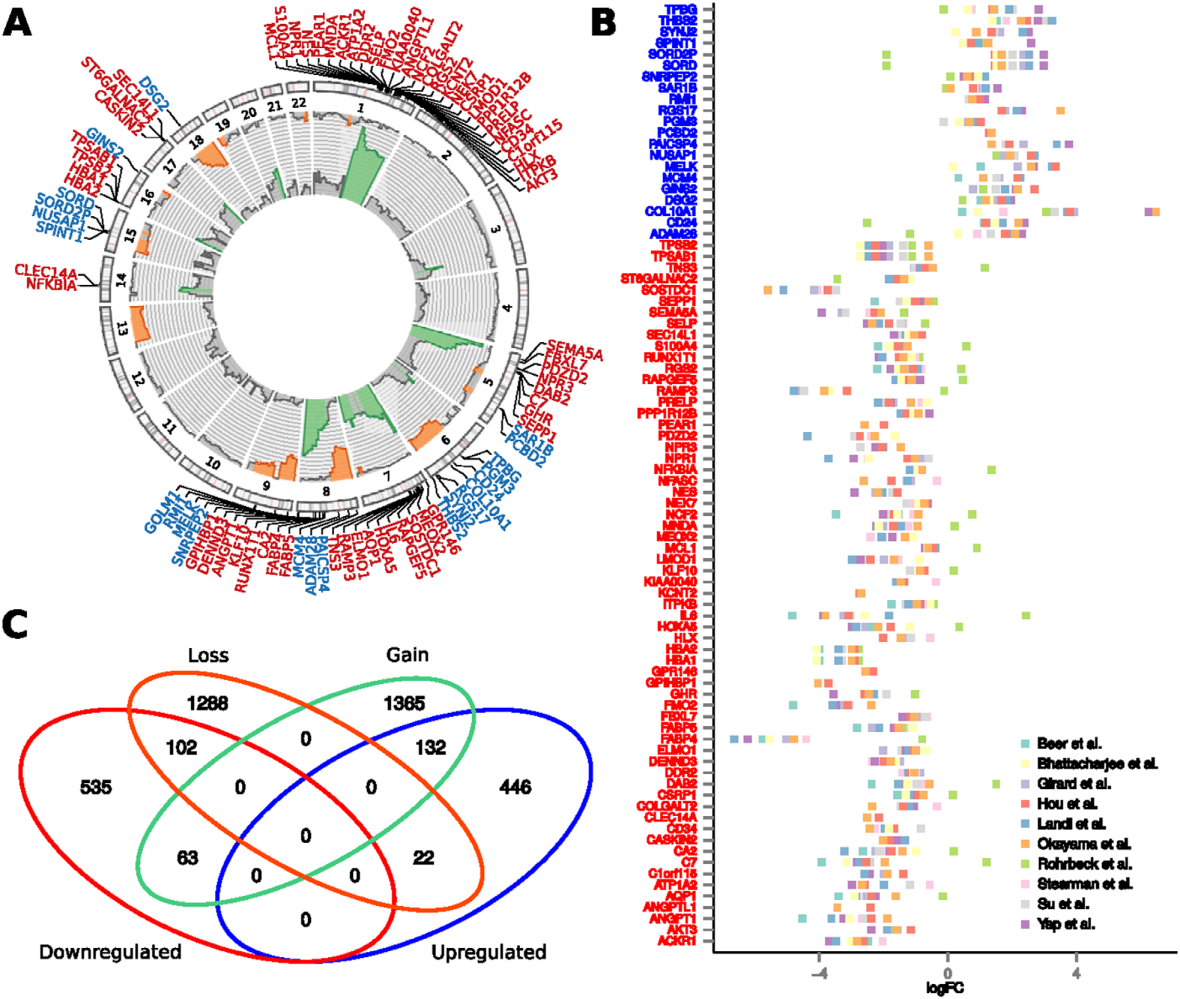
Association between chromosomal copy number aberrations and differential expression of genes **(A)** Frequencies of gains (pointing outbound) and losses (pointing inbound) of the given chromosomal region as obtained from integrative analysis of three aCGH datasets. The aberration frequencies are depicted in range from 0-50% and regions with significant frequency of aberrations are highlighted by color (orange – losses, green – gains). Precise chromosomal locations of these paradoxical genes are depicted in the circular plot. **(B)** Tumor-vs-normal expression fold change of the paradoxical genes, obtained across 10 publicly available datasets. In Figures A and B, symbols of the downregulated genes are labeled red, while the symbols of the upregulated genes are labeled blue. **(C)** Venn diagram showing overlaps between up-/downregulated genes and genes residing within the regions of chromosomal copy number gain, or loss.

First, we examined the frequency and statistical significance of autosomal CNAs across 3 LUAD aCGH datasets, including our new data and two publicly available datasets (see Materials and Methods). We identified multiple chromosomal regions with significantly (p < 0.05, randomization test) high frequency of gains (more than two copies) or losses (less than two copies); frequencies and corresponding p-values are listed in Supplementary Data 1. The most extensive positive aberrations identified occur on the q-arm of chromosomes 1, 7 and 8 as well the p-arm of chromosomes 5 and 7 (Figure 2A). The most significant copy number losses occurred on the q-arm of chromosomes 6, 9, 13, 15, and 18, along with the p-arm of chromosomes 8 and 9.

To identify genes whose differential expression remained consistent across patient cohorts, we performed integrative analysis of 10 publicly available gene expression datasets, comprising 740 LUAD samples and 241 normal tissue samples. Among 15,323 genes that were subjected to the robust rank analysis, we identified 1,309 genes that were significantly deregulated across the datasets (p < 0.01, robust rank aggregation), where 701 of these were downregulated genes, and 608 are upregulated. Excluding 9 non-protein-coding genes, reduced the numbers to 600 upregulated and 700 downregulated genes (see Supplementary Data 2). Non-protein coding genes involve downregulated *C17orf91*, and eight upregulated miRNA sequences: MIR7112, MIR6847, MIR7113, MIR671, MIR4647, MIR93, MIR25, MIR4721, all of which are intragenic miRNAs residing within upregulated genes. In subsequent meta-analysis of miRNA expression in LUAD, we found none of these miRNAs to be significantly deregulated.

We identified 132 (14.6%) downregulated genes residing in regions with decreased number of copies and 102 (22%) upregulated ones residing in regions with increased number of copies (p < 2.2E-16, Chi-squared test). Importantly, 63 consistently downregulated and 22 consistently upregulated genes reside on chromosomal regions with opposite direction of aberration – gains and losses, respectively (Figure 2). Hereafter, we refer to these 85 genes as paradoxical genes (Supplementary Table 1).

### Validation of the CNAs and differential expression of the paradoxical genes

While we identified paradoxical genes using data from diverse patient cohorts, we sought to validate our findings in an independent, homogeneous datasets. We used data from TCGA comprising: CNA, mRNA-seq and miRNA-seq LUAD data from 514 LUAD and 57 normal samples. This dataset was selected for validation solely on the basis of the CNA, mRNA and miRNA expression data availability, without considering clinicopathological characteristics of the data. Five of the 85 paradoxical genes could not be evaluated due to the missing CNA or expression data. From the remaining 80, we successfully validated 70 genes (p < 1E-4, randomization test), whose copy aberration status and differential expression confirmed results from the integrative analysis (Supplementary Figure 1, Supplementary Table 1). Further analysis only considers these 70 validated paradoxical genes.

**Figure 3:**
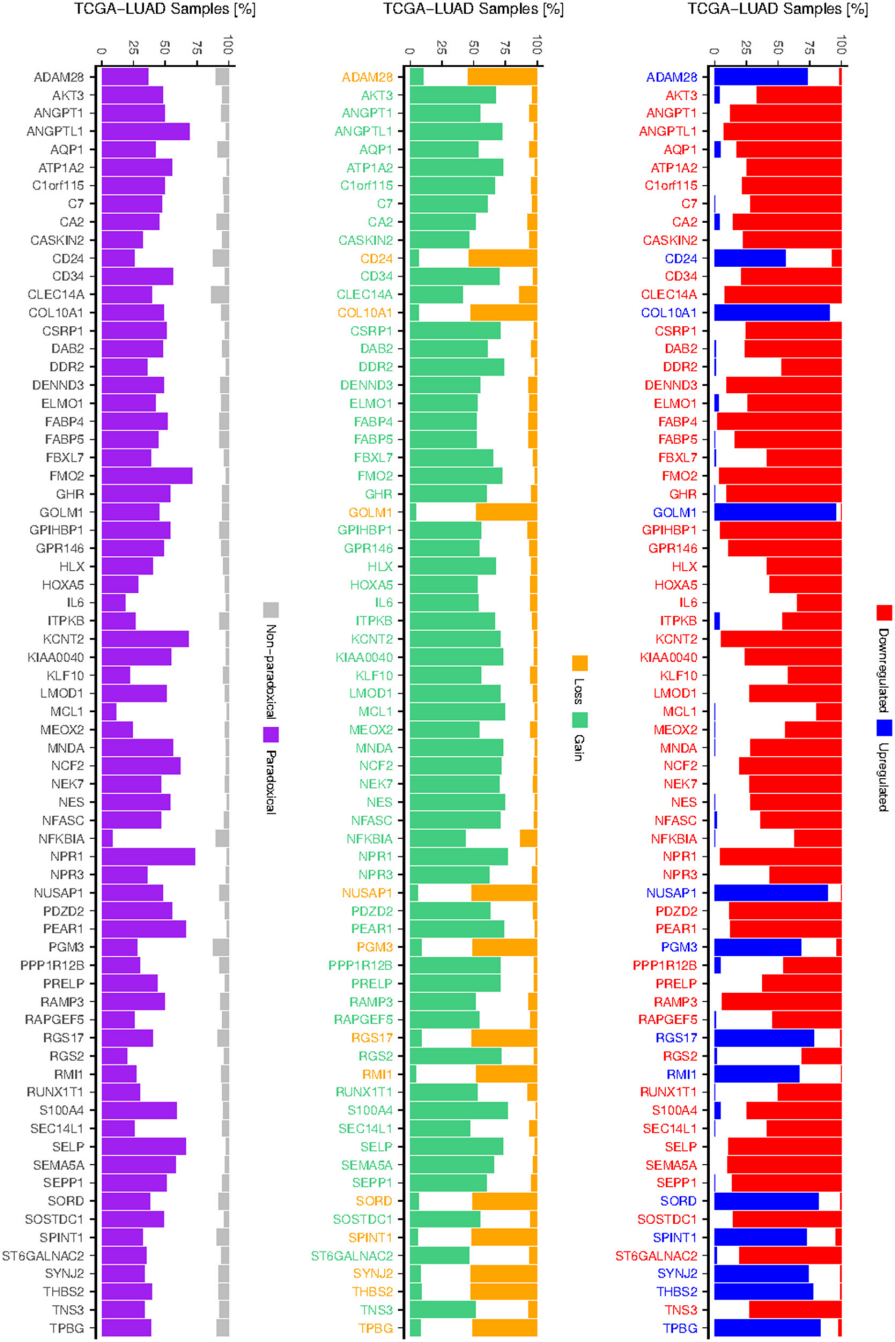
Frequency of deregulation and CNAs of paradoxical genes Barplot at the left shows frequencies of paradoxical and non-paradoxical co-occurrence of deregulated expression and CNAs. Barplots depicting frequencies of up- and downregulation (middle), gain and losses (right) of 70 validated paradoxical genes, as occur across TCGA LUAD samples. Colors of the gene labels indicate their deregulation/CNA status as obtained from the integrative analysis.

To test whether paradoxical deregulation occurs in the individual samples, we measured frequencies of paradoxical co-occurrence of up-/downregulation (expression z-score >/< +/−1.647) and losses/gains (log2 CNA >/< +/−0.2) of the paradoxical genes across individual TCGA LUAD samples (Figure 3). We found that frequency of paradoxical deregulation ranges from 9% (NFKBIA) to 74% (NPR1) of samples, median frequency equal to 46% and mean 44%. For all the 70 genes frequency of paradoxical deregulation exceeds the frequency of regular (non-paradoxical) deregulation.

### Co-occurrence between deregulated miRNAs and paradoxical genes

**Figure 4:**
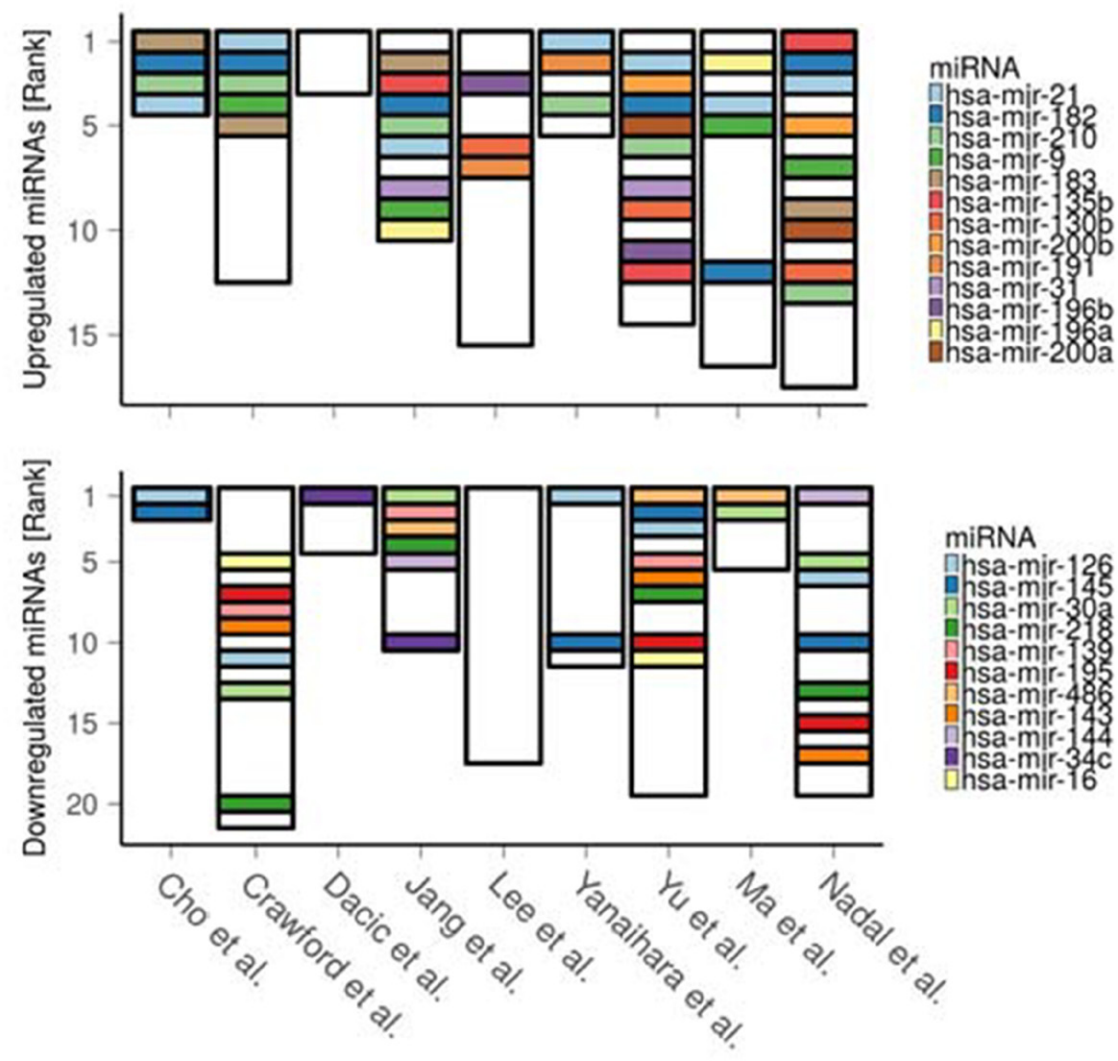
Ranking of the differentially expressed miRNAs as reported across 9 LUAD miRNA studies The lower the rank the greater the reported significance (and/or expression fold change) of the corresponding miRNA. Height of the bars denotes total number of reported miRNAs in each study.

We hypothesize that differential expression of paradoxical genes can be to a large extent explained by deregulation of the miRNAs that target these genes, either directly or through regulatory mediators, such as transcription factors. We thus performed meta-analysis of 9 papers reporting differentially expressed miRNAs in LUAD to identify consistently deregulated miRNAs. We found 24 such miRNAs (p < 0.05, robust rank analysis, see Methods section), 13 of which were upregulated (hsa-mir-21, hsa-mir-182, hsa-mir-210, hsa-mir-9, hsa-mir-183, hsa-mir-135b, hsa-mir-130b, hsa-mir-200b, hsa-mir-191, hsa-mir-31, hsa-mir-196b, hsa-mir-196a, hsa-mir-200a, ordered by significance) and 11 downregulated (hsa-mir-126, hsa-mir-145, hsa-mir-30a, hsa-mir-218, hsa-mir-139, hsa-mir-195, hsa-mir-486, hsa-mir-143, hsa-mir-144, hsa-mir-34c, hsa-mir-16) (Figure 4, Supplementary Data 3).

We then tested co-occurrence between the miRNAs deregulation and emergence of the paradoxical genes within the same cohort of patients. We first assessed differential expression of the 24 consistently deregulated miRNAs using TCGA-LUAD miRNA-seq data. Nineteen significantly deregulated miRNAs (out of 24, p = 1.18E-8, hypergeometric test) validated the results of the meta-analysis (hsa-mir-130b, hsa-mir-135b, hsa-mir-139, hsa-mir-143, hsa-mir-144, hsa-mir-182, hsa-mir-183, hsa-mir-195, hsa-mir-196a, hsa-mir-196b, hsa-mir-200a, hsa-mir-200b, hsa-mir-21, hsa-mir-210, hsa-mir-218, hsa-mir-30a, hsa-mir-31, hsa-mir-486, hsa-mir-9). While five miRNAs (hsa-mir-191, −34c, −126, −145, −16) did not validate; hsa-mir-191, -34c failed to pass the validation criteria only due to insufficient expression fold change, although their expression was altered significantly (Supplementary Table 2). Therefore, only 19 validated miRNAs are used for further analysis.

### Association between deregulated miRNAs and paradoxical genes

**Figure 5:**
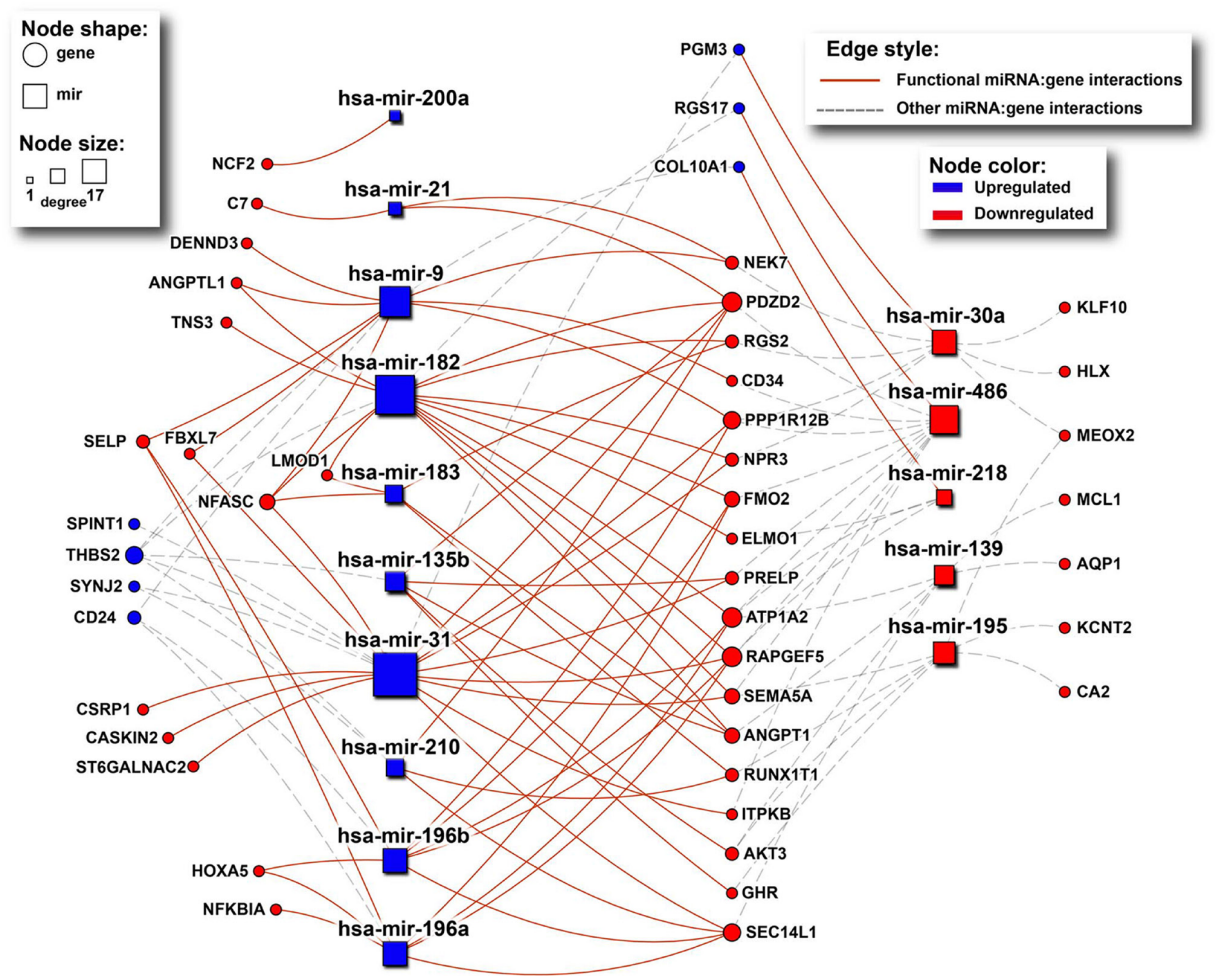
The network of interactions between deregulated miRNAs and their paradoxical gene targets as obtained from mirDIP Rectangles and circles represent miRNAs and genes, respectively. Red color denotes downregulated transcripts, while blue denotes upregulated ones. Size of nodes corresponds to number of interactions (degree). Solid red lines indicate miRNA:gene interactions between inversely deregulated transcripts, indicating potential causal associations.

According to mirDIP [21], 46 of the 70 paradoxical genes (65.7%) are targeted by subsets of the 19 deregulated miRNAs (p = 4.8E-3, hypergeometric test; Figure 5). Moreover, for 35 of these 46 targeted paradoxical genes, we found at least one deregulated miRNA that targets the given gene with its expression status contrasting the expression status of the given gene, implying that paradoxical expression of this gene could be explained by the miRNA deregulation.

**Figure 6:**
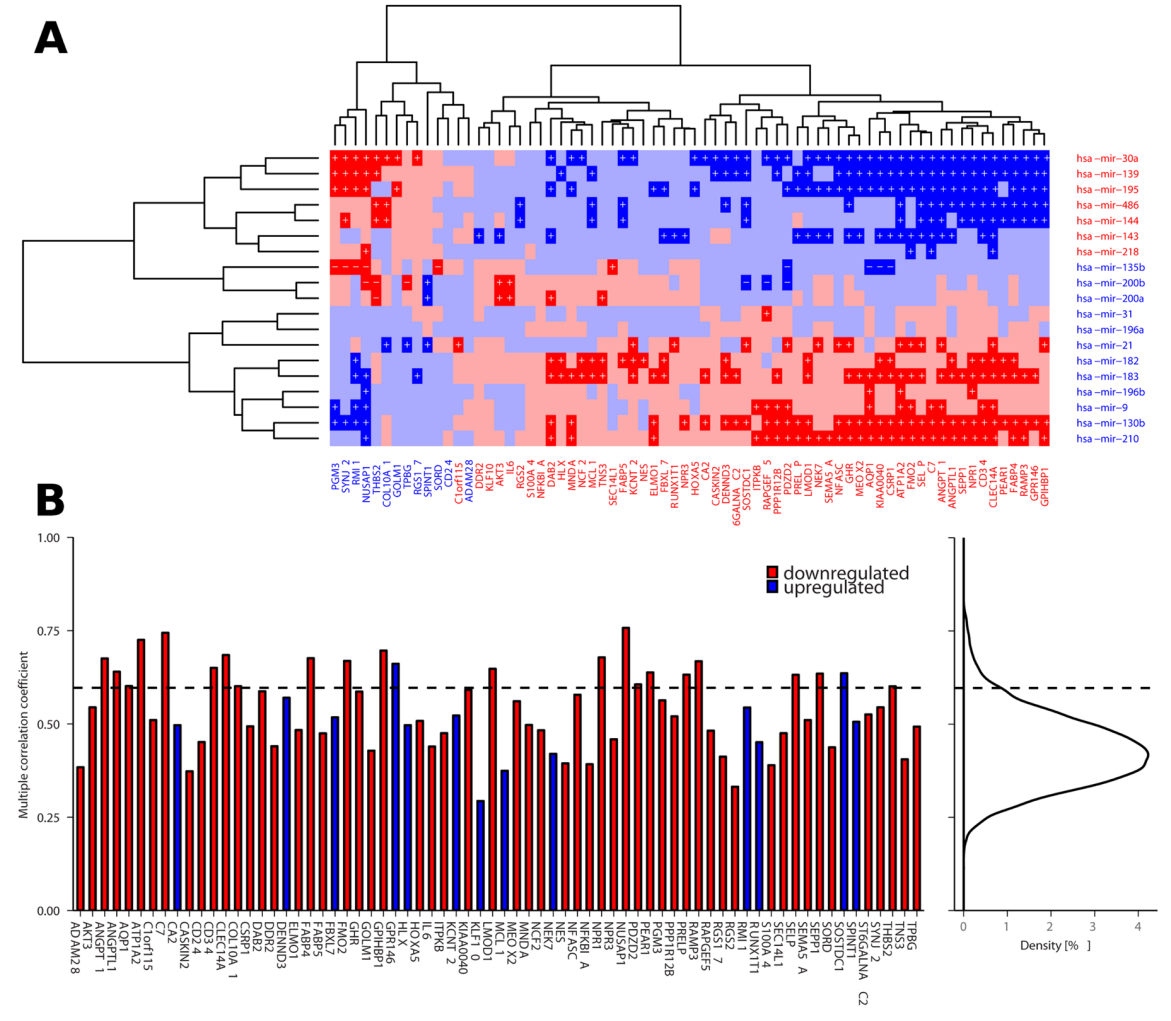
Correlation between deregulated miRNAs and paradoxical genes **(A)** Partial correlations between deregulated miRNAs and paradoxical genes as measured across TCGA LUAD data (red denotes negative correlation, blue denotes positive correlation, darker shade indicates significant correlations, p < 0.05). Plus signs denote partial correlation with causal explanation of gene deregulation, minus signs denote correlations that are significant but non-explanatory. **(B)** Barplot showing multiple correlations between paradoxical genes and *en block* deregulated miRNAs as calculated across TCGA LUAD data. Curve on the right depicts distribution of the same measure across all the genes in the TCGA LUAD data. Dashed line denotes 95th percentile of the distribution; there are 23 (32.9%) paradoxical genes whose multiple correlation coefficient falls among the top 5% of the highest values.

To further asses the association between expression of individual miRNAs and paradoxical genes we calculated partial correlation between them [22], using copy number status of the paradoxical genes as a controlling variable. We found 369 significantly co-expressed miRNA:gene pairs (27% of all miRNA:gene combinations), 362 (98.1%) of which are explanatory, i.e., there is a positive correlation between miRNAs and genes deregulated in the same direction, or negative correlation between inversely deregulated ones (Figure 6A). We found 64 paradoxical genes (91.4%) whose correlation with at least one of the 19 validated miRNAs is among the top 5% of the correlations measured between 10E+4 random pairs of genes and miRNAs, and whose paradoxical expression can be explained by deregulation of the upstream miRNA.

As downstream effects of the deregulation of individual miRNAs may be combined, we aimed to evaluate potential associations between expression of individual paradoxical genes and *en bloc* expression of the deregulated miRNAs. We calculated coefficients of multiple (multivariate) correlation (CMC) between the paradoxical genes and deregulated miRNAs. The value of CMC can be interpreted as the correlation between dependent variable (gene expression) and its best prediction that can be computed linearly from the set of independent variables (expression of miRNAs). We found 23 paradoxical genes (32.9%) with CMCs in the top 5% of the values of the same measure as calculated across 17,745 genes covered by TCGA-LUAD RNA-seq data (p = 1.7E-14, hypergeometric test; Figure 6B).

### Clinical significance of the paradoxical genes

**Figure 7:**
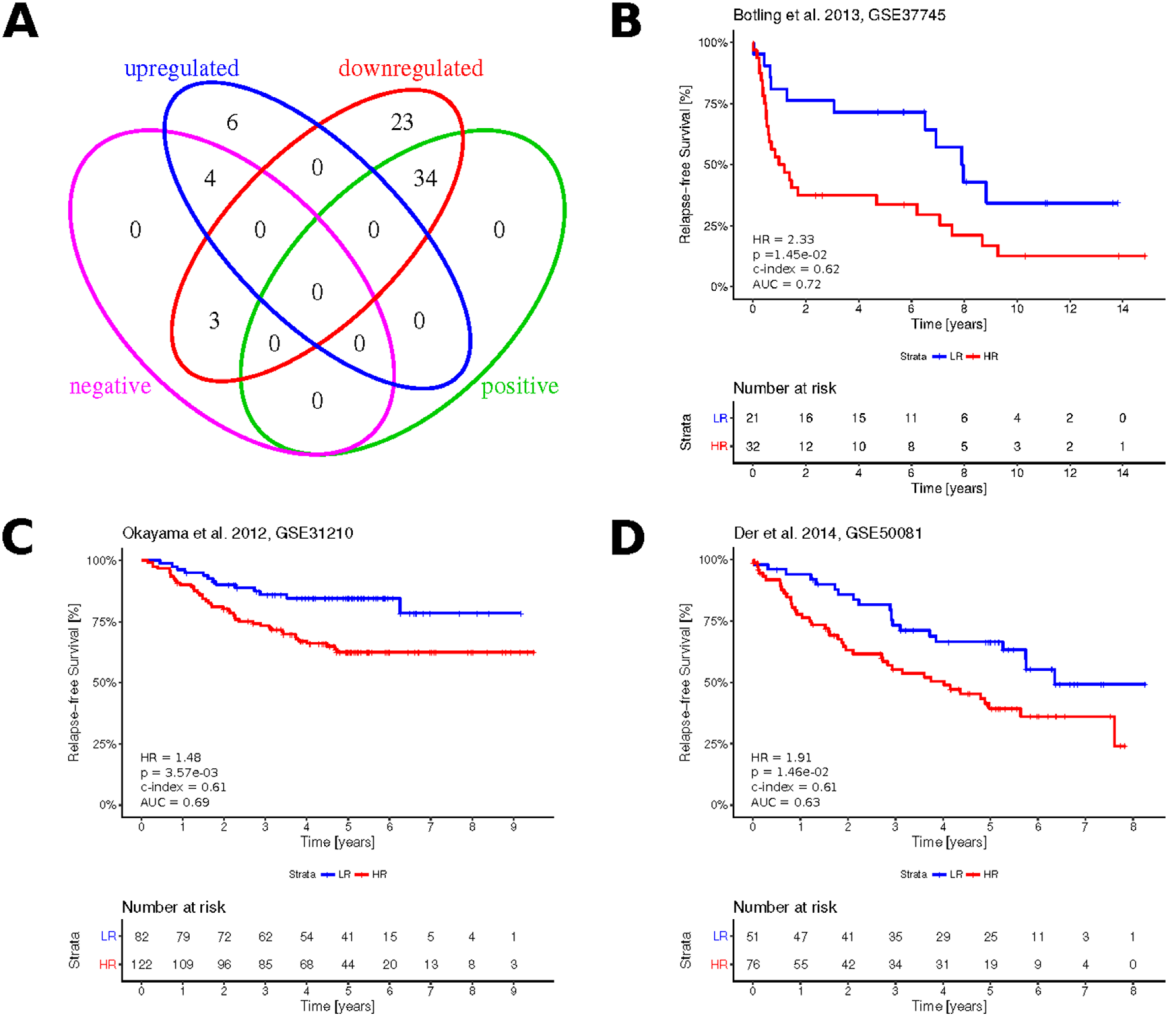
Clinical significance of the paradoxical genes **(A)** Venn diagram showing overlapping subsets of paradoxical genes with up-/ downregulated expression and subsets with positive (HR < 1, FDR < 0.05) and negative (HR > 1, FDR < 0.05) association with LUAD prognosis, as obtained from KMplot. **(B–D)** Kaplan-Meier plots showing survival curves in the three independent validation cohorts, as stratified based on the Cox proportional hazards calculated from paradoxical genes expression. Numbers in the bottom left, indicate resulting hazard ratio (HR), associated statistical significance of patient stratification (p), concordance index (c-index), area under ROC curve (AUC) calculated at five years.

Using KMplot [23], we assessed the association of the 70 paradoxical genes with patient disease-free survival. We found 41 (58.6%) of these genes as significantly associated with survival (FDR < 0.05). We assume that down-regulation of significantly positive genes (HR < 1, FDR < 0.05) as well as the up-regulation of significantly negative ones (HR > 1, FDR < 0.05) worsens the survival prognosis. Under this assumption, with the exception of three genes (ELMO1, DENND3, SPINT1), the actual deregulation of paradoxical genes is associated with a negative impact on patient survival (Figure 7A; Supplementary Table 3). This implies that the gene expression paradoxes we identified here mostly worsen the prognosis of LUAD patients.

Using TCGA-LUAD RNA-seq and matching clinical data, we constructed a multivariate Cox prognostic model, where expressions of the paradoxical genes served as prognostic variables. The model was validated using three independent publicly available gene expression datasets and associated clinical data: Botling *et al.* [24], Okayama *et al.* [25] and Der *et al.* [26] (Figures 7B-7D). The resulting concordance index, area under ROC curve, hazard ratio between risk groups and associated P-value, demonstrated robust prognostic potential of paradoxical genes signature.

### Pathway enrichment analysis of the paradoxical genes

To elucidate biological functions of the paradoxical genes we performed a comprehensive pathway enrichment analysis. Using Pathway Data Integration Portal (pathDIP) [27] we identified 22 pathways significantly enriched by the 70 paradoxical genes (FDR < 0.05, hypergeometric test). A list of all pathways and respective gene memberships is provided in Supplementary Data 4. Interestingly, several of the enriched pathways are related to lipid metabolism and signaling (adipogenesis, LPA receptor mediated events, regulation of lypolysis) are key players in carcinogenesis [28]. Moreover, several enriched guidance molecule pathways (ephrin signaling, semaphorin interactions, integrin, DCC-mediated attractive signaling) are noted as cancer-drug targets [29]. PTEN-dependent cell cycle arrest, apoptosis pathway as well as hemostasis are known to play a role in cancer [30–32].

## DISCUSSION

Integrating three LUAD aCGH datasets, we identified several chromosomal regions with extensive copy number aberrations. Weir and colleagues [33] reported similar profile of copy number aberrations in 371 LUAD samples, confirming gains on 1q, 5p, 7p, 7q and 8q as well as deletions on 6q, 8p, 9p, 9q, 13q, 18q (Table 1). Lee *et al.* [34] obtained similar results using Molecular Inversion Probe assays on 12 LUAD samples, confirming gains on 1q, 5p, 7p, 7q and 8q and losses on 6q, 8p, 18q (but not losses on 9p, 9q, 13q and 15q). While the individual aberrations vary greatly among individuals, as even the most frequent aberrations appear only in less than 50% of samples, the overall CNA profile of LUAD is conserved across the patient cohorts.

**Table 1:** List of the large scale copy number aberrations in LUAD

CNA	Arm	Frequency [%]	Weir et al., 2007 [33]	Lee et al., 2012 [34]
Gain:	1q	30-45	x	x
	5p	22-45	x	x
	7p	19-34	x	x
	7q	16-20	x	x
	8q	15-35	x	x
Loss:	6q	7-13	x	x
	8p	7-26	x	x
	9p	13-20	x	
	9q	7-12	x	
	13q	7-12	x	
	15q	7-8	x	
	18q	8-21	x	x

Frequency indicates range of relative number of aberrations occurring across the span of the given region. Checkmarks indicate aberrations confirmed by the above studies.

By integrative analysis of multiple gene expression and copy number datasets, we found significant association between CNA status and differential expression of genes. Similar associations were previously reported in other cancer types [2–5, 7]. However, we also discovered 85 paradoxical genes whose expression was in opposite direction to their CNA. Seventy of these genes were subsequently validated across a homogeneous LUAD data cohort from TCGA. Paradoxical expression of these genes was validated even on the individual samples, proving that such paradoxical gene expression is a well preserved feature of the molecular profile of LUAD.

Expression of paradoxical genes is associated with miRNAs consistently deregulated across multiple LUAD patient cohorts. Deregulation of these miRNAs inverts the effects of the genomic aberrations occurring in tumors. This is demonstrated by significant overlap between paradoxical genes and targets of these miRNAs, as well as by the two methods we applied here to measure correlation between the paradoxical genes and given miRNAs. Although correlation does not imply causality, the results of our analysis strongly suggest that differential expression of paradoxical genes is caused by deregulation of miRNAs.

We tested prognostic relevance of the paradoxical genes and found 41 (58.6%) paradoxical genes significantly associated with patient survival. Paradoxical expression of 39 genes has negative impact on prognosis. We also developed paradoxical gene signature that was validated on a three independent validation datasets [24–26].

While the majority of the paradoxical genes are novel and their association with LUAD prognosis has not been investigated thoroughly, there are 17 validated paradoxical genes that have been previously associated with prognosis of other cancers: *ADAM28* [35, 36], *ANGPT1* [37], *CA2* [38], *CA24* [39], *DAB2* [40], *HOXA5* [41, 42], *IL6* [43], *KLF10* [44], *MCL1* [45], *NES* [46], *NUSAP1* [47], *PDZD2* [48], *RGS17* [49, 50], *RUNX1T1* [51, 52], *SELC14L1* [53], *SEMA5A* [54] and *SEPP1* [55] (Table 2).

**Table 2:** List of validated paradoxical genes whose association with cancer prognosis has previously been reported

Gene	Association to cancer prognosis	Ref.
ADAM28	Overexpression correlates with cell proliferation and lymph node metastasis	[35]
	Serological and histochemical marker for NSCLC	[36]
ANGPT1	Role in the prognosis of patients with oral squamous cell cancer	[37]
CA2	Downregulated in gastric cancer and proposed as an independent prognostic factor for patient survival	[38]
CD24	Expression at early stages of breast cancer indicates a highly invasive tumor	[39]
DAB2	An important tumor suppressor, frequently downregulated in various tumors	[40]
HOXA5	Downregulation is associated with poor prognosis in NSCLC	[41]
	Shown to prevent tumor progression and metastasis in colon cancer	[42]
IL6	SNP associated with risk of multiple cancers	[43]
KLF10	Potential clinical predictor for progression of pancreatic cancer	[44]
MCL1	Key molecule for acquiring epithelial-to-mesenchymal transition-associated chemo-resistance in NSCLC	[45]
NES	Marker of cancer stem cells	[46]
NUSAP1	Encodes protein that is proposed biomarker for prostate cancer recurrence	[47]
PDZD2	Shown to induce senescence or quiescence of prostate, breast and liver cancer cells via transcriptional activation of p53	[48]
RGS17	Induces tumor cell proliferation lung and prostate cancers	[49]
	Regulator of cell survival and chemoresistance in ovarian cancer	[50]
RUNX1T1	Predictor of liver metastasis in pancreatic endocrine tumours	[51]
	Associated with proliferation and senescence inhibition in t(8;21)-positive leukaemic cells	[52]
SELC14L1	Proposed marker for predicting prognosis and progression of prostate cancer	[53]
SEMA5A	Over-expressed pancreatic cancer cells, regulates tumorigenesis, proliferation, invasion and metastasis, and serve as a target for diagnosis and treatment of pancreatic cancer	[54]
SEPP1	Inversely related to pancreatic cancer risk	[55]

From the panel of consistently deregulated miRNAs, several were recently associated with cancer progression and prognosis. Most notably, miRNAs hsa-mir-21 and -196b are well established oncomirs [56], and hsa-mir-196a is known to be associated with NSCLC [57]. The role of hsa-mir-30a varies across human cancer types [58]. Hsa-mir-135b reverses chemoresistance of NSCLC [59]. Hsa-mir-139 is associated with aggressive tumor behavior and disease progression in breast cancer [60], and is believed to inhibit bladder cancer proliferation and self-renewal [61]. Hsa-mir-143 inhibits tumor growth of breast cancer [62]. Hsa-mir-144 has been shown to induce cell cycle arrest and apoptosis in pancreatic cancer cells [63]. Hsa-mir-182 promotes prostate cancer progression [64]. Hsa-mir-195 inhibits the proliferation and invasion of pancreatic cancer cells [65]. Hsa-mir-218 downregulation contributes to epithelial-mesenchymal transition and tumor metastasis in lung cancer [66]. Hsa-mir-130b is a part of the new prognostic marker for patient risk assessment and as an indicator of therapy resistance in prostate cancer [67]. Similarly, hsa-mir-183 in combination with hsa-mir-19b were recently proposed as biomarkers of lung cancer [68], and miRNAs hsa-mir-145 and -9 as biomarkers for early-stage cervical cancer [69].

Genomic instability is one of the cancer hallmarks [70] that results in CNAs and differential expression of various genes. However, paradoxical genes with expression patterns opposite to gene dosage status often are dismissed as noise and overlooked in genome-wide cancer gene discovery efforts [8]. Prognostic significance of the paradoxical genes suggests that their deregulation in LUAD is crucial for cancer progression and is maintained by the cancer cells despite the CNAs affecting expression of these genes in an inverse manner. We found that deregulation of the paradoxical genes is maintained at the epigenetic level by a group of deregulated miRNAs. These findings highlight the importance of integrative analysis, which combines information across diverse types of high-throughput data.

## MATERIALS AND METHODS

### Copy number aberrations: sample collection, preparation and data processing

The samples used in this study were from the banked resected tumors collected in the BR.10 adjuvant chemotherapy trial [71]. The study has received approval by the Institutional Research Ethics Board. A total of 142 formalin-fixed paraffin embedded (FFPE) and 16 snap-frozen samples were included. Haematoxylin and eosin stained slides from FFPE blocks first were reviewed by a lung pathologist to locate tumor rich areas (tumor cellularity > 60%), and then the block was cored at this area. Cored specimens were de-paraffinized by incubation in xylene overnight, and then washed with ethanol and air dried. Qiagen ATL buffer (QIAamp^©^ DNA extraction kit cat. 51306, Germantown, MD) were added. Specimens were digested by proteinase K at 55°C overnight at 450rpm (Eppendorf^©^ Thermomixer R, Fisher Scientific). DNA isolation followed the manufacturer’s protocol (Qiagen, Cat. 51306, Germantown, MD). Samples of isolated genomic DNA were quantified by Nanodrop 1000 (Thermo Scientific, Wilmington, DE) and electrophoresed in 0.8% agarose gel to visualize DNA size distribution. Severely degraded samples (80% of DNA fragments with size < 20bp) were excluded. Eight out of 142 FFPE and none of the snap-frozen samples were excluded. The final cohort contains 134 FFPE and 16 snap-frozen samples.

Test and reference DNA were labeled using Cy3 and Cy5 dCTPs respectively; 200 ng of genomic DNA was labeled using the BioPrime DNA labeling system (Invitrogen). Prior to hybridization, test and reference labeled DNA were combined and purified using a ProbeQuant Sephadex G-50 Column (Amersham, GE Healthcare Life Sciences, Chicago, IL) to remove unincorporated nucleotides. Then 100 μg of Human Cot-1 DNA (Invitrogen) was added to the labeled sample prior to precipitation with 0.1 volume 3M sodium acetate and 2.5 volumes of ethanol. The DNA pellet was resuspended in 20 μl DIG Easy hybridization solution (Roche, Indianapolis, IN), 2.5 μl (20 μg/μl) sheared herring sperm DNA and 2.5 μl (100μg/μl) yeast tRNA (Calbiochem, San Diego, CA). DNA was denatured at 85°C for 10 minutes and repetitive sequences were blocked at 37°C for one hour prior to hybridization.

Prehybridization was carried out using 20 μl DIG Easy hybridization buffer (Roche), 2.5 μl 10% BSA and 2.5 μl (20 μg/μl) sheared herring sperm DNA, at 45°C for 1 hour. Hybridization was carried out at 45°C for 24-48 hours. Arrays were washed for 5 × 5 min., in 0.1 x SSC, 0.1% SDS at room temperature in the dark with agitation. Each array was then rinsed 5 times in a clean slide box containing 0.1 x SSC with agitation. Slides were then dried with (oil free) nitrogen air stream and stored in the dark until imaging.

Array image capture and data normalization were performed as previously described [72]. Briefly, post-hybridization arrays were scanned using a CCD-based imaging system (Virtek ChipReader), and quantitated using Soft-Worx Tracker spot analysis software (Applied Precision, Issaquah, WA).

Data were log2 transformed, and replicate clones having standard deviations > 0.075 or signal-to-noise ratios in each dye channel of < 3 were filtered out. A multi-step normalization was then performed to control for biases caused by the array (e.g., spatial biases or differences in background signal), the dyes used for labeling, or the DNA sample quality [73, 74]. The amount of “copycat'” correction required for each sample was plotted in a histogram of all samples; those that required too much correction and did not lie within a normal distribution were deemed to be poor quality DNA, and were eliminated from analysis. By these criteria, 35 samples were eliminated, leaving 115 samples (including 63 LUAD samples used here) from 113 patients for further analysis. Data from all 115 samples are publicly accessible through: http://ophid.utoronto.ca/aCGH/.

### Analysis of the copy number aberrations data

In addition to our newly-produced CNA data, we analyzed two publicly available aCGH datasets acquired from LUAD tumor samples (see Table 3). Each of the public datasets was first normalized, segmented and additionally underwent post-segmentation normalization using methods provided by Bioconductor package CGHcall (v2.22.0) [75]. All three datasets then underwent a “calling” process using the CGHcall method from the same package, converting the continuous log-ratios on each probe, to one of the three discrete values (calls) corresponding to: (i) decreased number of copies (loss), (ii) normal copy and (iii) increased number of copies (gain) [75].

**Table 3:** Summary of the public datasets used in this study

	Author & Year	No. of samples (total/normal)	Source & Notes
CNAs:	Chitale et al., 2009 [93]	199	http://cbio.mskcc.org/public/
	Job et al., 2010 [97]	60	E-TABM-926, ArrayExpress
Gene expression:	Bhattacharjee et al., 2001 [98]	207/17	http://www.broadinstitute.org/MPR/lung/
	Beer et al., 2002 [99]	96/10	GSE68571
	Stearman et al., 2005 [100]	39/19	GSE2514
	Yap et al., 2005 [101]	58/9	E-MEXP-231, ArrayExpress
	Su et al., 2007 [102]	54/27	GSE7670
	Landi et al., 2008 [103]	107/50	GSE10072
	Rohrbeck et al., 2008 [85]	15/5	GSE6044
	Hou et al., 2010 [86]	109/64	GSE19188
	Girard et al., 2011	50/20	GSE31547, Unpublished
	Okayama et al., 2012 [25]	246/20	GSE31210
miRNA expression:	Yanaihara et al., 2006 [87]	208/104	
	Cho et al., 2009 [88]	20/10	
	Crawford et al., 2009 [89]	20/8	
	Dacic et al., 2010 [90]	12/6	
	Yu et al., 2010 [91]	40/20	
	Lee et al., 2011 [92]	12/6	
	Jang et al., 2012 [94]	206/103	
	Ma et al., 2014 [95]	108/54	
	Nadal et al., 2014 [96]	101/10	

As the individual datasets come with different probesets, obtained copy numbers calls correspond to different chromosomal segments and cannot be compared directly. We then integrated results acquired from individual datasets by assigning a set of chromosomal positioning “anchors” that comprised the starts and ends of chromosomal locations of all the probes in the four datasets, as described by Guo et al. [76]. Then for each anchor, if the anchor was within the chromosomal location of the probe from any of the datasets, acquired vector of states corresponding to the probe were assigned to this anchor. Conversely, if an anchor was outside of any of the probes of the given dataset, a vector of missing values was created and assigned to the anchor. Anchors with missing values from more than one datasets were removed. Number of losses and gains were calculated across the anchors and their statistical significance was evaluated by p-values, calculated by comparing actual gains/losses counts to those obtained from 10^6^ permutations.

### Analysis of gene expression datasets

We analyzed 10 publicly available gene expression datasets (Table 1) which satisfied the following criteria: (i) were originally from studies on tissue samples from surgically resected human LUAD tumors, (ii) contained at least one sample of noncancerous normal tissue for comparison, and (iii) were produced by using Affymetrix platforms to enable uniform processing and analysis of all the datasets. We first normalized and summarized each dataset by Gene Chip Robust Multiarray Averaging (gcrma, v2.38.0) [77]. For each individual dataset, we then evaluated differential expression of the genes using Bioconductor package limma (v3.32.7) [78]. Based on the expression fold change, genes were classified as either up- or downregulated, and then ranked according to statistical significance, which was evaluated by FDR-adjusted p-value. Analyzing 10 datasets resulted in 10 rankings for upregulated genes and 10 for downregulated ones. To identify consistently deregulated genes, obtained rankings were subjected to robust rank aggregation analysis implemented in R package RobustRankAggreg (v1.1) [79]. This analysis detects genes that are ranked consistently better than expected under the null hypothesis of uncorrelated inputs, and assigns a p-value as a significance score for each gene. The stability of the resulting significance scores was assessed by the leave-one-out correction, in which the same analysis was repeated 10 times, each time excluding one of the rankings. Acquired p-values from each round were averaged into a corrected p-value. Genes whose significance score was smaller than chosen threshold (corrected p < 0.01) were further considered as consistently significantly deregulated genes.

### Meta-analysis of the miRNA expression

Compared to gene expression studies, fewer miRNA expression profiles from LUAD are available, and various platforms are used, often including custom arrays. Therefore, to provide analysis of miRNA expression in LUAD, instead of acquiring and processing expression data, we summarized reported results of 9 published miRNA expression studies (Table 1). Full text and (if applicable) supplementary data of each of the studies were carefully examined, and miRNAs with significantly altered expression were selected for further analysis. miRNA names were standardized according to miRBase (release 21) [80]. All miRNAs were classified as either up- or downregulated and ranked according to their reported statistical significance. If this was not reported, expression fold change was used instead. Examining 9 studies we obtained 9 rankings for upregulated miRNAs and 9 for downregulated ones. Analogously to gene expression analysis described in the previous section, obtained miRNA rankings subsequently were subjected to the robust rank aggregation analysis and leave-one-out correction of the obtained p-values. miRNAs whose significance score was smaller than a chosen threshold (corrected p < 0.05), comprised the resulting list of consistently significantly deregulated miRNAs.

### Acquiring chromosomal locations and copy number status of the deregulated genes

Using Bioconductor package biomaRt (v2.22) [81] we determined the chromosomal locations of the deregulated genes from the Ensembl (v75, Feb. 2014) database and compared these locations with chromosomal coordinates of the aberrant regions. Deregulated genes whose chromosomal locations overlapped with aberrant regions were counted and statistical significance of the association between the aberrations and differential gene expression was then evaluated using Chi-square test.

### Identification of the miRNA-target pairs

We used microRNA Data Integration Portal, v2.3.2.0 (mirDIP; http://ophid.utoronto.ca/mirDIP) [21] to acquire data on human miRNAs and their respective targets. mirDIP integrates data from 14 miRNA resources and supports a search for miRNA-target pairs under user-defined filters, including a number of independent confirmations of given pairs, confidence criteria, etc. We restricted our search to only miRNA-target pairs that fell among the top third of the most confident predictions from at least two different sources. miRNA names were standardized as described above, and symbols of their gene targets were standardized by HGNC symbol checker (http://www.genenames.org, version from September 2015). To assess the significance of overlap between targets of deregulated miRNAs and paradoxical genes, we performed hypergeometric testing, using 15,323 genes that were subjected to robust rank analysis as a population and 7,836 of these genes that, according to mirDIP are targeted by at least one of the deregulated miRNAs as a number of “successes” in the population.

### Calculation of the miRNA:target partial correlation and multiple correlation coefficients

Partial correlation coefficients between gene and miRNA expressions were calculated using R package ppcor v1.1, using copy number of the given gene as a third – controlling variable. Statistical significance of the obtained values was calculated by two-sided comparison with distribution of the same measure obtained across 10^4^ random miRNA:gene pairs. To distinguish whether correlations between miRNA and given genes may also imply causal association, we compared the sign of the correlation and copy number status of the miRNA and gene. If the miRNA and gene were deregulated in the same direction, mutual correlation must be positive to indicate a causal association. If the miRNA and genes were inversely deregulated, mutual correlation must be negative to indicate causal association.

Multiple correlation coefficients between gene and *en bloc* miRNA expression *C* was calculated as follows:

*C* ^2^ = *c^T^R*^−1^*c*

where *c* denotes a vector of Pearson coefficients of correlation between a given genes and miRNA expressions, *R* denotes a matrix of Pearson coefficients of correlation between miRNA expressions.

### Evaluation of the prognostic significance of the paradoxical genes

Prognostic properties of the individual genes were evaluated by KMplot (http://kmplot.com/analysis/) [23], version 2015, using only LUAD patient data and corresponding disease-free survival censored at 10 years. If multiple probe sets were mapped to the same gene, we used only JetSet probes mapping to a given gene. Obtained hazard ratios (HR) and associated p-values were then summarized and multiple testing adjustment of the p-values was subsequently computed using false discovery rate (FDR) method.

To evaluate the multivariate prognostic potential of the paradoxical genes we developed a Cox proportional hazards model, where expressions of 70 validated paradoxical genes served as covariates. The model was derived using R package glmnet [82] (v2.0.2), applying LASSO (L1) regularization to prevent over-fitting. TCGA-LUAD RNA-seq data were standardized by converting to z-scores and along with the corresponding clinical data were used as “training data”. The resulting model was then validated on three independent datasets, and its predictive performance was first evaluated by a concordance index (function survConcordance from R package survival [83], v2.38.3), and an area under receiver operating characteristics curve (AUC), measured at the fifth year after initial time point (function AUC.cd from the R survAUC package, v 1.0.5). Patients were then separated into two groups based on the predicted risk score, using its 40^th^ percentile as a threshold. This threshold was selected based on ROC analysis of the model using training data. Validated HR between these two groups, as well as associated statistical significance (log-rank test) were calculated (function survdiff from the survival package) and Kaplan-Meier survival curves of both groups were plotted (for more details see [84]).

### Pathway enrichment analysis

Using Pathway Data Integration Portal v2.5.1.2 (http://ophid.utoronto.ca/pathDIP), we performed comprehensive pathway enrichment analysis across 20 major pathway databases [27]. We considered literature curated gene:pathway memberships as well as those predicted according to experimentally detected protein-protein interactions (including interactions experimentally detected between orthologues plus FpClass interactions with minimum confidence level for predicted associations equal 0.95; for more details see pathDIP documentation).

## SUPPLEMENTARY MATERIALS FIGURE AND TABLES













## Abbreviations

AUC: area under receiver operating characteristics
aCGH: array comparative genomic hybridization
CMC: coefficient of multiple correlation
CNAs: copy number aberrations
FDR: false discovery rate
FFPE: formalin-fixed paraffin-embedded
GCRMA: gene chip robust multiarray averaging
HR: hazard ratio
LUAD: lung adenocarcinoma
TCGA: the cancer genome atlas.
