# Supplementary material for: Differentially expressed microRNAs in lung adenocarcinoma invert effects of copy number aberrations of prognostic genes
Source: Oncotarget. 2018 Jan 8;9(10):9137–55. doi: 10.18632/oncotarget.24070 (PMC5823624; doi:10.18632/oncotarget.24070)
Supplement: Supplementary file 1 [file oncotarget-09-9137-s001.pdf]

Differentially expressed microRNAs in lung adenocarcinoma invert effects of copy number aberrations of prognostic genes

SUPPLEMENTARY MATERIALS

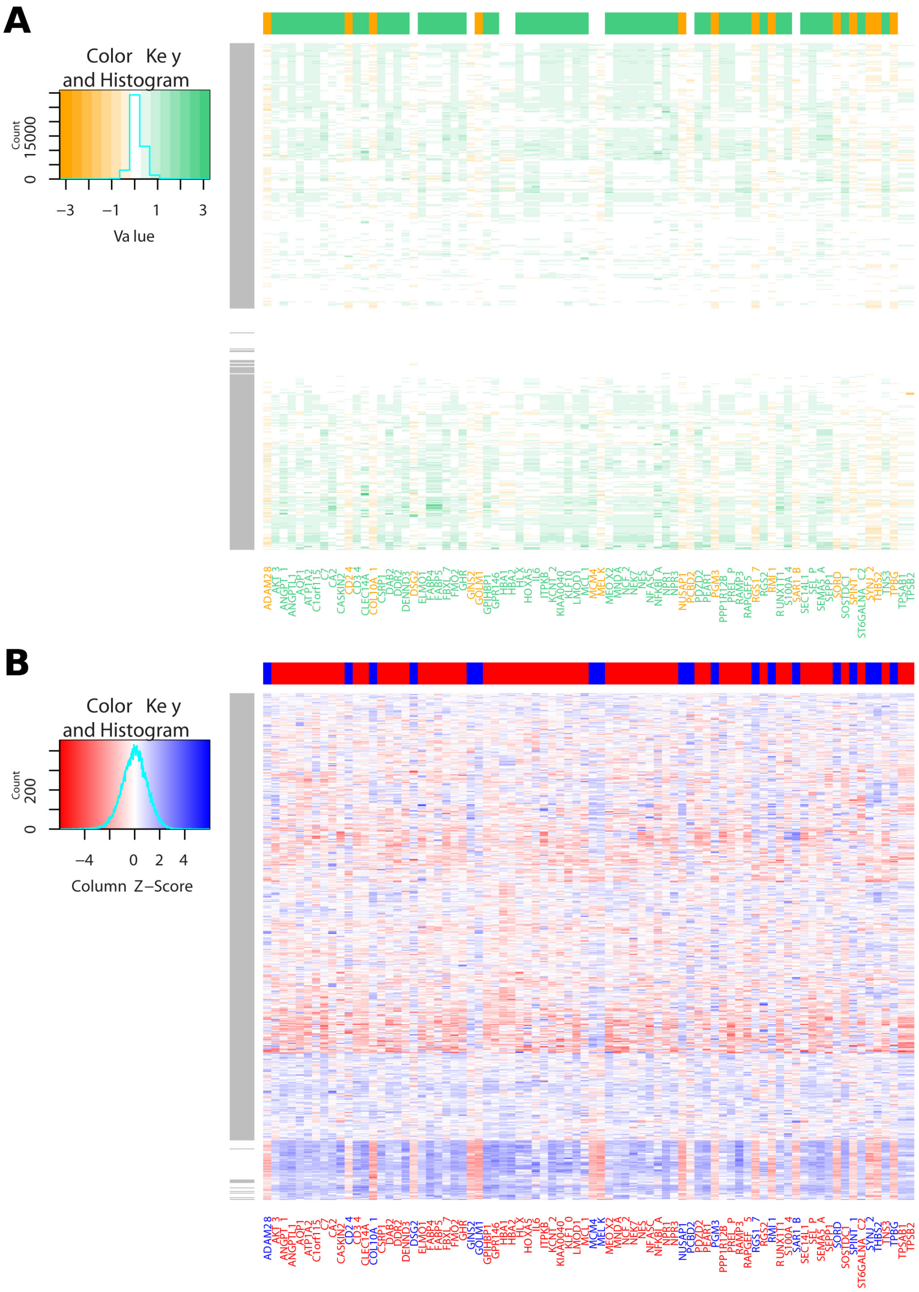

Supplementary Figure 1: Copy number **(A)** and expression **(B)** of the paradoxical genes across TCGA LUAD data. Expression depicted are z-scores calculated across samples. Vertical bar indicates histology of the sample, gray color corresponds to tumor samples and white to normal tissue samples. Horizontal bar indicates resulting copy number and expression status of the genes. Orange corresponds to copy number loss and green corresponds to gain. Blue and red correspond to up- and downregulation, respectively. White color corresponds to normal copy number, or expression status. Identical color notation was applied to gene labels, where color denotes copy number or expression status of the gene as obtained by integrative analysis.

Supplementary Table 1: List of paradoxical genes with their chromosomal location, along with robust rank score and expression status as obtained from integrative gene expression analysis

See Supplementary File 1

Supplementary Table 2: List of deregulated miRNAs along with their expressions obtained from TCGA miRNAseq data

miRNA	Integrative analysis		TCGA expression:	
	Corr. score	Status	logFC	Adj. P-val.
hsa-mir-126	2.91E-07	down	-0.03	8.63E-01
hsa-mir-130b*	2.07E-03	up	1.86	8.00E-29
hsa-mir-135b*	1.15E-03	up	2.94	1.08E-29
hsa-mir-139*	9.36E-04	down	-1.87	4.82E-25
hsa-mir-143*	3.72E-03	down	-1.96	2.20E-24
hsa-mir-144*	1.83E-02	down	-2.56	2.08E-21
hsa-mir-145	2.38E-05	down	-0.59	1.04E-03
hsa-mir-16	4.85E-02	down	0.62	4.33E-06
hsa-mir-182*	3.19E-09	up	2.69	1.19E-53
hsa-mir-183*	1.59E-05	up	2.52	1.08E-43
hsa-mir-191	2.02E-02	up	0.95	1.07E-12
hsa-mir-195*	3.22E-03	down	-1.58	1.16E-27
hsa-mir-196a*	3.58E-02	up	3.49	1.26E-12
hsa-mir-196b*	3.43E-02	up	2.13	2.12E-08
hsa-mir-200a*	4.14E-02	up	2.29	4.54E-34
hsa-mir-200b*	1.67E-02	up	1.20	4.31E-11
hsa-mir-21*	8.32E-14	up	2.92	6.24E-85
hsa-mir-210*	5.59E-09	up	5.26	3.30E-59
hsa-mir-218*	1.39E-04	down	-1.34	1.66E-22
hsa-mir-30a*	4.00E-05	down	-1.83	5.63E-22
hsa-mir-31*	3.42E-02	up	2.62	4.05E-12
hsa-mir-34c	3.74E-02	down	-0.97	6.26E-03
hsa-mir-486*	8.16E-03	down	-3.57	2.48E-39
hsa-mir-9*	9.67E-06	up	4.49	1.50E-30

Asterisks next to a miRNA name indicates miRNAs validated in the TCGA LUAD data.

Supplementary Table 3: Evaluation of the prognostic properties of the 70 validated paradoxical genes

See Supplementary File 1

Supplementary Data 1: Frequencies and statistical significance of the CNAs as obtained from the integrative analysis of the three aCGH datasets

See Supplementary File 2

Supplementary Data 2: Results of the robust-rank integrative analysis of the gene expression datasets

See Supplementary File 3

Supplementary Data 3: Results of the robust-rank meta-analysis of the miRNA expression reports

See Supplementary File 4

Supplementary Data 4: Results of the pathway enrichment analysis as obtained from pathDIP

See Supplementary File 5
