# Supplementary material for: Differentially expressed microRNAs in lung adenocarcinoma invert effects of copy number aberrations of prognostic genes
Source: Oncotarget. 2018 Jan 8;9(10):9137–55. doi: 10.18632/oncotarget.24070 (PMC5823624; doi:10.18632/oncotarget.24070)
Supplement: Supplementary file 2 [file oncotarget-09-9137-s002.doc]

**Supplementary Tables**

**Supplementary Table 1:** List of paradoxical genes with their chromosomal location, along with robust rank score and expression status as obtained from integrative gene expression analysis.

| Gene symbol | Chromosomal location: | | | Integrative analysis: | | TCGA expression: | | TCGA CNAs: | |
| --- | --- | --- | --- | --- | --- | --- | --- | --- | --- |
|  | Chr. | Start | End | Corr. score | Status | logFC | FDR. | logFC | FDR |
| ADAM28* | 8 | 24294040 | 24359018 | 8.0E-04 | up | 2.11 | 7.2E-26 | -0.17 | 8.2E-35 |
| CD24* | 6 | 106969831 | 106975627 | 2.7E-03 | up | 1.15 | 2.0E-08 | -0.15 | 4.0E-39 |
| COL10A1* | 6 | 116118923 | 116158747 | 1.3E-08 | up | 4.78 | 3.8E-62 | -0.14 | 6.9E-41 |
| DSG2 | 18 | 31498043 | 31549008 | 1.3E-04 | up | 1.20 | 4.5E-15 | -0.06 | 2.7E-02 |
| GINS2 | 16 | 85676198 | 85690073 | 6.1E-03 | up | 2.86 | 1.3E-53 | -0.07 | 5.3E-08 |
| GOLM1* | 9 | 86026146 | 86100173 | 7.2E-13 | up | 2.57 | 3.7E-69 | -0.12 | 1.0E-40 |
| MCM4 | 8 | 47960185 | 47978160 | 3.3E-03 | up | 2.05 | 1.4E-40 | 0.14 | 1.1E-25 |
| MELK | 9 | 36572862 | 36677683 | 3.5E-04 | up | 3.84 | 3.9E-58 | -0.10 | 1.7E-16 |
| NUSAP1* | 15 | 41332694 | 41381050 | 1.8E-04 | up | 2.58 | 6.3E-53 | -0.13 | 1.2E-37 |
| PAICSP4 | 8 | 4787332 | 4788584 | 4.1E-08 | up |  |  |  |  |
| PCBD2 | 5 | 134904906 | 135007959 | 5.9E-03 | up | 0.26 | 1.5E-03 | -0.07 | 1.2E-11 |
| PGM3* | 6 | 83161150 | 83193936 | 9.8E-03 | up | 0.73 | 1.4E-15 | -0.14 | 3.0E-37 |
| RGS17* | 6 | 153004459 | 153131249 | 1.9E-03 | up | 3.44 | 2.5E-32 | -0.14 | 6.1E-37 |
| SNRPEP2 | 9 | 6748703 | 6748981 | 2.3E-04 | up |  |  |  |  |
| SORD* | 15 | 45023104 | 45077185 | 4.3E-10 | up | 1.37 | 7.5E-31 | -0.12 | 1.1E-37 |
| SORD2P | 15 | 44825747 | 44884694 | 4.2E-10 | up |  |  |  |  |
| RMI1* | 9 | 83980711 | 84004074 | 9.1E-03 | up | 0.93 | 5.5E-25 | -0.12 | 1.5E-36 |
| SAR1B | 5 | 134601144 | 134649271 | 2.2E-03 | up | 0.80 | 6.0E-22 | -0.07 | 8.1E-12 |
| SPINT1* | 15 | 40844018 | 40858207 | 3.3E-03 | up | 0.82 | 5.5E-15 | -0.13 | 5.9E-38 |
| SYNJ2* | 6 | 157981887 | 158099176 | 6.8E-04 | up | 1.61 | 6.2E-35 | -0.15 | 6.9E-39 |
| THBS2* | 6 | 169215780 | 169254044 | 1.8E-07 | up | 2.67 | 9.3E-34 | -0.15 | 1.3E-38 |
| TPBG* | 6 | 82363206 | 82370828 | 1.7E-04 | up | 1.71 | 1.6E-32 | -0.14 | 1.0E-38 |
| ACKR1 | 1 | 159203307 | 159206500 | 7.1E-06 | down |  |  |  |  |
| AKT3* | 1 | 243488233 | 243851079 | 1.7E-05 | down | -1.23 | 3.8E-16 | 0.22 | 1.1E-78 |
| ANGPT1* | 8 | 107249482 | 107498055 | 1.2E-07 | down | -2.73 | 9.7E-38 | 0.20 | 3.1E-43 |
| ANGPTL1* | 1 | 178849705 | 178871052 | 4.3E-03 | down | -3.11 | 7.7E-51 | 0.23 | 3.6E-97 |
| AQP1* | 7 | 30911855 | 30925516 | 2.6E-03 | down | -2.57 | 5.4E-23 | 0.17 | 1.5E-46 |
| ATP1A2* | 1 | 160115759 | 160143591 | 6.7E-03 | down | -3.02 | 2.1E-27 | 0.27 | 1.3E-91 |
| C1orf115* | 1 | 220689845 | 220699157 | 2.7E-04 | down | -1.82 | 4.8E-25 | 0.21 | 8.6E-76 |
| C7* | 5 | 40909252 | 40982939 | 1.6E-04 | down | -2.71 | 1.2E-17 | 0.25 | 1.4E-58 |
| CA2* | 8 | 85463852 | 85481493 | 7.4E-03 | down | -2.35 | 6.4E-27 | 0.19 | 1.6E-37 |
| CASKIN2* | 17 | 75500261 | 75515583 | 6.9E-06 | down | -1.53 | 6.8E-42 | 0.12 | 6.4E-37 |
| CD34* | 1 | 207880972 | 207911402 | 1.7E-05 | down | -1.59 | 1.0E-37 | 0.23 | 4.5E-86 |
| CLEC14A* | 14 | 38254103 | 38256369 | 4.9E-03 | down | -2.29 | 4.1E-60 | 0.17 | 3.6E-15 |
| COLGALT2 | 1 | 183929854 | 184037729 | 5.9E-03 | down |  |  |  |  |
| CSRP1* | 1 | 201483530 | 201509456 | 1.2E-06 | down | -0.89 | 1.1E-31 | 0.23 | 7.0E-92 |
| DAB2* | 5 | 39371675 | 39462300 | 4.1E-03 | down | -1.20 | 2.1E-20 | 0.25 | 3.3E-54 |
| DDR2* | 1 | 162631373 | 162787400 | 8.4E-04 | down | -1.34 | 4.2E-12 | 0.26 | 1.5E-93 |
| DENND3* | 8 | 141117278 | 141195808 | 1.4E-08 | down | -1.87 | 2.9E-54 | 0.21 | 1.8E-47 |
| ELMO1* | 7 | 36854361 | 37449249 | 1.8E-03 | down | -1.18 | 1.9E-17 | 0.17 | 6.2E-45 |
| FABP4* | 8 | 81478419 | 81483263 | 2.2E-09 | down | -6.13 | 1.7E-73 | 0.21 | 5.3E-36 |
| FABP5* | 8 | 81280363 | 81284777 | 2.3E-04 | down | -2.31 | 1.4E-30 | 0.21 | 7.4E-36 |
| FBXL7* | 5 | 15500196 | 15939795 | 1.9E-03 | down | -1.13 | 5.4E-16 | 0.28 | 1.7E-67 |
| FMO2* | 1 | 171185208 | 171211230 | 9.7E-08 | down | -3.66 | 9.6E-55 | 0.24 | 8.1E-98 |
| GHR* | 5 | 42423777 | 42721878 | 2.2E-05 | down | -2.51 | 1.3E-39 | 0.24 | 7.4E-56 |
| GPIHBP1* | 8 | 143213193 | 143217170 | 1.9E-03 | down | -4.45 | 8.4E-62 | 0.20 | 1.6E-47 |
| GPR146* | 7 | 1044576 | 1059261 | 5.6E-03 | down | -2.25 | 7.7E-61 | 0.18 | 1.1E-44 |
| HBA1 | 16 | 176680 | 177522 | 4.3E-07 | down | -6.02 | 3.9E-40 | 0.03 | 6.7E-01 |
| HBA2 | 16 | 172847 | 173710 | 4.2E-07 | down | -4.72 | 7.1E-57 | 0.03 | 6.7E-01 |
| HLX* | 1 | 220879400 | 220885059 | 2.0E-04 | down | -1.31 | 1.9E-25 | 0.21 | 3.7E-78 |
| HOXA5* | 7 | 27141052 | 27143668 | 3.1E-07 | down | -1.70 | 5.3E-26 | 0.17 | 2.5E-45 |
| IL6* | 7 | 22725884 | 22732002 | 2.3E-03 | down | -2.40 | 2.6E-20 | 0.19 | 3.4E-47 |
| ITPKB* | 1 | 226631690 | 226739323 | 3.9E-03 | down | -0.60 | 1.7E-07 | 0.21 | 8.4E-79 |
| KCNT2* | 1 | 196225779 | 196609225 | 5.3E-03 | down | -3.49 | 8.7E-57 | 0.23 | 3.8E-93 |
| KIAA0040* | 1 | 175156987 | 175192999 | 2.2E-05 | down | -1.17 | 1.0E-33 | 0.24 | 3.6E-98 |
| KLF10* | 8 | 102648779 | 102655902 | 1.2E-03 | down | -0.95 | 2.0E-22 | 0.21 | 5.8E-53 |
| LMOD1* | 1 | 201896452 | 201946588 | 2.1E-05 | down | -1.94 | 7.3E-29 | 0.23 | 2.5E-86 |
| MCL1* | 1 | 150574551 | 150579738 | 4.2E-03 | down | -0.47 | 2.7E-09 | 0.30 | 4.1E-89 |
| MEOX2* | 7 | 15611212 | 15686812 | 2.8E-03 | down | -1.44 | 5.7E-13 | 0.19 | 8.0E-48 |
| MNDA* | 1 | 158831317 | 158849506 | 3.1E-03 | down | -1.71 | 1.1E-20 | 0.26 | 1.2E-91 |
| NCF2* | 1 | 183555563 | 183590876 | 2.7E-03 | down | -1.80 | 4.6E-30 | 0.23 | 5.1E-95 |
| NEK7* | 1 | 198156963 | 198322420 | 1.8E-04 | down | -0.94 | 1.1E-29 | 0.23 | 1.4E-88 |
| NES* | 1 | 156668763 | 156677397 | 1.6E-04 | down | -1.70 | 7.9E-26 | 0.27 | 6.6E-97 |
| NFASC* | 1 | 204828651 | 205022822 | 9.5E-03 | down | -1.75 | 2.8E-17 | 0.23 | 1.4E-88 |
| NFKBIA* | 14 | 35401511 | 35404749 | 4.8E-03 | down | -0.87 | 1.0E-13 | 0.17 | 1.1E-17 |
| NPR1* | 1 | 153678637 | 153693992 | 1.7E-04 | down | -3.06 | 3.4E-51 | 0.31 | 1.2E-80 |
| NPR3* | 5 | 32689070 | 32791724 | 2.7E-03 | down | -1.88 | 1.8E-18 | 0.26 | 4.2E-62 |
| PDZD2* | 5 | 31639410 | 32110931 | 7.0E-05 | down | -2.80 | 1.6E-37 | 0.26 | 1.4E-64 |
| PEAR1* | 1 | 156893698 | 156916434 | 4.6E-03 | down | -2.18 | 2.8E-42 | 0.27 | 3.4E-94 |
| PPP1R12B* | 1 | 202348699 | 202592706 | 6.9E-03 | down | -0.75 | 2.4E-07 | 0.23 | 5.4E-92 |
| PRELP* | 1 | 203475828 | 203491352 | 2.9E-03 | down | -1.76 | 8.3E-19 | 0.23 | 3.0E-90 |
| RAMP3* | 7 | 45157791 | 45186302 | 3.3E-09 | down | -3.10 | 2.9E-73 | 0.17 | 3.6E-43 |
| RAPGEF5* | 7 | 22118238 | 22357144 | 1.6E-03 | down | -1.01 | 1.2E-13 | 0.19 | 4.2E-49 |
| RGS2* | 1 | 192809039 | 192812283 | 9.5E-03 | down | -0.94 | 9.3E-09 | 0.23 | 1.4E-94 |
| RUNX1T1* | 8 | 91954967 | 92103286 | 1.9E-03 | down | -1.66 | 3.6E-16 | 0.19 | 5.3E-41 |
| S100A4* | 1 | 153543613 | 153550136 | 1.6E-05 | down | -1.54 | 2.6E-17 | 0.29 | 2.4E-98 |
| SEC14L1* | 17 | 77086716 | 77217101 | 3.2E-06 | down | -0.98 | 1.6E-20 | 0.13 | 1.4E-39 |
| SELP* | 1 | 169588849 | 169630193 | 1.5E-06 | down | -2.66 | 1.5E-35 | 0.24 | 1.5E-97 |
| SEMA5A* | 5 | 9035026 | 9546075 | 1.4E-06 | down | -2.96 | 4.4E-49 | 0.28 | 9.5E-68 |
| SEPP1* | 5 | 42799880 | 42887392 | 3.4E-03 | down | -1.93 | 4.8E-31 | 0.24 | 1.8E-57 |
| SOSTDC1* | 7 | 16461481 | 16530580 | 2.8E-06 | down | -5.39 | 6.8E-42 | 0.19 | 2.1E-49 |
| ST6GALNAC2* | 17 | 76563710 | 76586956 | 8.1E-05 | down | -2.05 | 3.4E-21 | 0.13 | 2.5E-37 |
| TNS3* | 7 | 47275154 | 47582558 | 3.4E-03 | down | -1.13 | 5.2E-25 | 0.17 | 5.4E-41 |
| TPSAB1 | 16 | 1240696 | 1242554 | 2.7E-03 | down | -2.17 | 7.9E-20 | 0.04 | 2.1E-01 |
| TPSB2 | 16 | 1227272 | 1230184 | 2.7E-03 | down | -2.25 | 9.7E-19 | 0.03 | 1.0 |

LogFC and FDR indicate tumor-vs.-normal expression log2 fold change and corresponding adjusted p-value as obtained from TCGA LUAD mRNAseq. Last two column to the right list log2 of the copy-number changes (as compared to ploidy) and corresponding adjusted p-value as obtained from TCGA LUAD CNA data. Asterisk next to a gene symbol indicates validation of the gene's paradoxical status in the TCGA LUAD data.

**Supplementary Table 3:** Evaluation of the prognostic properties of the 70 validated paradoxical genes.

| Gene | Probe | HR | P-value | FDR | Signature coeff. |
| --- | --- | --- | --- | --- | --- |
| CSRP1 | 200621_at | 0.66 | 8.90E-03 | 1.89E-02 | 0.080 |
| SORD | 201563_at | 2.06 | 7.10E-06 | 5.52E-05 | 0.079 |
| NUSAP1 | 218039_at | 2.24 | 7.10E-07 | 9.94E-06 | 0.079 |
| NFASC | 213438_at | 0.49 | 8.90E-06 | 5.66E-05 | -0.075 |
| FMO2 | 228268_at | 0.45 | 1.20E-06 | 1.20E-05 | -0.067 |
| ANGPTL1 | 239183_at | 0.6 | 2.00E-03 | 5.00E-03 | 0.066 |
| RUNX1T1 | 242845_at | 0.57 | 5.90E-04 | 2.17E-03 | -0.065 |
| MCL1 | 200797_s_at | 0.57 | 4.30E-04 | 1.67E-03 | 0.060 |
| C7 | 200797_s_at | 1.26 | 1.45E-01 | 2.07E-01 | -0.051 |
| ATP1A2 | 203296_s_at | 0.42 | 8.70E-08 | 2.03E-06 | 0.050 |
| PRELP | 228224_at | 0.42 | 1.70E-07 | 2.98E-06 | -0.044 |
| NPR3 | 219789_at | 0.79 | 1.29E-01 | 1.94E-01 | 0.040 |
| DAB2 | 201278_at | 0.53 | 7.10E-05 | 3.11E-04 | 0.036 |
| SEPP1 | 201427_s_at | 0.49 | 8.30E-06 | 5.66E-05 | -0.036 |
| FBXL7 | 213249_at | 0.68 | 1.50E-02 | 2.76E-02 | 0.035 |
| DDR2 | 225442_at | 0.63 | 5.10E-03 | 1.19E-02 | -0.035 |
| IL6 | 205207_at | 1.13 | 4.40E-01 | 5.22E-01 | 0.027 |
| PDZD2 | 209493_at | 0.41 | 2.80E-08 | 9.80E-07 | -0.023 |
| GHR | 205498_at | 0.56 | 2.20E-04 | 9.06E-04 | -0.019 |
| MNDA | 204959_at | 0.79 | 1.40E-01 | 2.04E-01 | -0.018 |
| LMOD1 | 203766_s_at | 0.5 | 1.70E-05 | 9.15E-05 | -0.008 |
| NEK7 | 212530_at | 0.6 | 1.30E-03 | 3.96E-03 | 0.007 |
| S100A4 | 203186_s_at | 1.2 | 2.50E-01 | 3.30E-01 | -0.006 |
| TNS3 | 217853_at | 0.74 | 6.20E-02 | 9.86E-02 | -0.004 |
| ADAM28 | 205997_at | 0.91 | 5.60E-01 | 6.13E-01 |  |
| AKT3 | 212609_s_at | 0.59 | 8.60E-04 | 3.01E-03 |  |
| ANGPT1 | 205609_at | 0.5 | 1.70E-05 | 9.15E-05 |  |
| AQP1 | 209047_at | 0.7 | 2.33E-02 | 4.08E-02 |  |
| C1orf115 | 218546_at | 0.88 | 4.10E-01 | 4.95E-01 |  |
| CA2 | 209301_at | 0.85 | 3.00E-01 | 3.82E-01 |  |
| CASKIN2 | 221846_s_at | 1.15 | 3.80E-01 | 4.67E-01 |  |
| CD24 | 266_s_at | 0.74 | 5.70E-02 | 9.28E-02 |  |
| CD34 | 209543_s_at | 0.79 | 1.30E-01 | 1.94E-01 |  |
| CLEC14A | 226244_at | 0.59 | 1.70E-03 | 4.58E-03 |  |
| COL10A1 | 217428_s_at | 0.97 | 8.70E-01 | 8.83E-01 |  |
| DENND3 | 212974_at | 1.62 | 2.60E-03 | 6.28E-03 |  |
| ELMO1 | 204513_s_at | 1.46 | 1.70E-02 | 3.05E-02 |  |
| FABP4 | 203980_at | 0.79 | 1.50E-01 | 2.10E-01 |  |
| FABP5 | 202345_s_at | 0.96 | 7.91E-01 | 8.24E-01 |  |
| GOLM1 | 217771_at | 1.12 | 4.60E-01 | 5.37E-01 |  |
| GPIHBP1 | 238062_at | 0.59 | 1.60E-03 | 4.48E-03 |  |
| GPR146 | 228770_at | 0.69 | 2.50E-02 | 4.27E-02 |  |
| HLX | 214438_at | 0.67 | 1.10E-02 | 2.14E-02 |  |
| HOXA5 | 213844_at | 0.61 | 2.00E-03 | 5.00E-03 |  |
| ITPKB | 1554306_at | 0.47 | 5.20E-06 | 4.55E-05 |  |
| KCNT2 | 244455_at | 0.66 | 1.04E-02 | 2.08E-02 |  |
| KIAA0040 | 203144_s_at | 1.04 | 8.00E-01 | 8.24E-01 |  |
| KLF10 | 202393_s_at | 0.76 | 8.50E-02 | 1.32E-01 |  |
| MEOX2 | 206201_s_at | 0.37 | 8.20E-10 | 5.74E-08 |  |
| NCF2 | 209949_at | 0.91 | 5.56E-01 | 6.13E-01 |  |
| NES | 218678_at | 0.82 | 2.00E-01 | 2.74E-01 |  |
| NFKBIA | 201502_s_at | 1.09 | 5.70E-01 | 6.14E-01 |  |
| NPR1 | 32625_at | 0.6 | 1.50E-03 | 4.38E-03 |  |
| PEAR1 | 32625_at | 0.49 | 2.20E-05 | 1.10E-04 |  |
| PGM3 | 210041_s_at | 1.18 | 3.00E-01 | 3.82E-01 |  |
| PPP1R12B | 201957_at | 0.51 | 2.40E-05 | 1.12E-04 |  |
| RAMP3 | 205326_at | 1.01 | 9.50E-01 | 9.50E-01 |  |
| RAPGEF5 | 204681_s_at | 0.45 | 1.00E-06 | 1.17E-05 |  |
| RGS17 | 220334_at | 1.07 | 6.50E-01 | 6.89E-01 |  |
| RGS2 | 202388_at | 0.66 | 8.70E-03 | 1.89E-02 |  |
| RMI1 | 218979_at | 1.39 | 3.92E-02 | 6.53E-02 |  |
| SEC14L1 | 202084_s_at | 0.6 | 1.30E-03 | 3.96E-03 |  |
| SELP | 206049_at | 0.66 | 1.04E-02 | 2.08E-02 |  |
| SEMA5A | 213169_at | 0.86 | 3.41E-01 | 4.27E-01 |  |
| SOSTDC1 | 213456_at | 0.83 | 2.29E-01 | 3.09E-01 |  |
| SPINT1 | 202826_at | 1.67 | 1.30E-03 | 3.96E-03 |  |
| ST6GALNAC2 | 204542_at | 0.91 | 5.35E-01 | 6.13E-01 |  |
| SYNJ2 | 212828_at | 1.1 | 5.56E-01 | 6.13E-01 |  |
| THBS2 | 203083_at | 1.49 | 1.33E-02 | 2.52E-02 |  |
| TPBG | 203476_at | 1.54 | 6.80E-03 | 1.54E-02 |  |

Hazard ratios (HR) and corresponding P-values were evaluated by KMplot (<http://kmplot.com/analysis/>) [23], and FDR adjusted (FDR). Signature coefficients were obtained for individual genes by fitting Cox proportional hazards model to TCGA LUAD mRNAseq data with respect to associated clinical information. Missing signature coefficient indicates that the gene was excluded from the signature due to the applied regularization.
